# Crystal structure, Hirshfeld surface analysis and DFT study of 2,2′′-({[(1*E*,1′*E*)-(diselanedi­yl)bis­(2,1-phenyl­ene)]bis­(methane­ylyl­idene)}bis­(aza­neylyl­idene))bis­[3′,6′-bis­(di­ethyl­amino)-4a’,9a’-di­hydro­spiro­[isoindoline-1,9′-xanthen]-3-one]

**DOI:** 10.1107/S2056989021013189

**Published:** 2022-01-01

**Authors:** Manzoor Ahmad Malla, Ravi Bansal, Ray J. Butcher, Sushil K. Gupta

**Affiliations:** a School of Studies in Chemistry, Jiwaji University, Gwalior 474011, India; b Department of Chemistry, Howard University, 525 College Street NW, Washington, DC 20059, USA

**Keywords:** Spiro­bicyclic diselenide, crystal structure, density functional theory (DFT), Hirshfeld surface analysis, two-dimensional fingerprint plots

## Abstract

The title compound, C_70_H_70_N_8_O_4_Se_2_, is a spiro bicyclic diselenide, made up of two [SeC_6_H_4_CH=N—N(CO)C_6_H_4_(C)C_6_H_3_NEt_2_(O)C_6_H_3_NEt_2_] units related by a twofold crystallographic symmetry element bis­ecting the diselenide bond. The compound crystallizes in a non-centrosymmetric polar space group (tetra­gonal, *P*  *b*2) and the structure was refined as an inversion twin. The two diethyl amine groups and their attached phenyl groups of the xanthene ring are disordered over two orientations, with occupancies of 0.664 (19)/0.336 (19) and 0.665 (11)/0.335 (11), respectively. The dihedral angles between the mean planes of the central isoindoline and the phenyl rings are 26.8 (2) and 2.5 (4)°, respectively. The mean plane of the central xanthene ring forms dihedral angles of 2.0 (5), 8.8 (5), 1.7 (5) and 7.9 (6)° with the peripheral phenyl rings. The isoindoline and xanthene rings subtend a dihedral angle of 89.8 (2)°. The mol­ecular conformation is stabilized by an intra­molecular C—H⋯O hydrogen bond generating an *S*(6) ring motif. In the crystal, mol­ecules are linked by C—H⋯O hydrogen bonds together with C—H⋯π (ring) inter­actions, forming a three-dimensional network. A Hirshfeld surface analysis of the crystal structure indicates that the most important contributions to the crystal packing are from H⋯H (68.1%), C⋯H/H⋯C (21.2%) and O⋯H/H⋯O (8.7%) contacts. The optimized structure calculated using density functional theory (DFT) at the B3LYP/6 – 31 G(*d*) level is compared with the experimentally determined mol­ecular structure in the solid state. The HOMO–LUMO behaviour was used to determine the energy gap and the mol­ecular electrostatic potential (MEP) of the compound was investigated.

## Abstract 2

The X-ray crystal and mol­ecular structures, DFT study and Hirshfeld surface analysis of a novel spiro­bicyclic diselenide are reported.

## Chemical context

Diaryl diselenides and aryl seleno­lates have been previously used as ligand precursors for the synthesis of transition-metal complexes (Khandelwal & Gupta, 1989 ▸; Gupta & Parihar, 1995 ▸, 1998 ▸; Gupta *et al.*, 1998 ▸). Seleno­spiro­cyclic compounds are a class of heterocyclic compounds with a wide variety of uses in organic synthesis (Aho *et al.*, 2005 ▸; Kotha *et al.*, 2009 ▸; James *et al.*, 1991 ▸), biological activities (Mugesh *et al.*, 2001 ▸; Nogueira *et al.*, 2004 ▸; Press *et al.*, 2008 ▸; Alberto *et al.*, 2009 ▸) and photoluminescence properties (Singh *et al.*, 2011 ▸; Shi *et al.*, 2010 ▸). However, the formation of spiro­bicyclic diselenides is rare and to the best of our knowledge, not reported in the literature. There are very few reports of the formation of seleno­spiro­cyclic derivatives which have been structurally characterized (Singh *et al.*, 2011 ▸; Shi *et al.*, 2010 ▸). Very recently, organoselenium compounds containing both N and Se have been reported with inter­esting intra- and inter­molecular inter­actions (Saravanan *et al.*, 2021 ▸). Although the synthetic and structural studies of various diselenides (see section 4, *Database survey*) are known in the literature, to the best of our knowledge, a synthesis and structural data have not yet been published for the title compound. Herein we report the crystal structure, DFT and Hirshfeld surface analysis of 2,2′′-({[(1*E*,1′*E*)-(diselanedi­yl)bis­(2,1-phenyl­ene)]bis­(methane­ylyl­idene)}bis­(aza­neylyl­idene))bis­[3′,6′-bis­(di­ethyl­amino)-4a’,9a’-di­hydro­spiro­[isoindoline-1,9′-xanthen]-3-one], isolated from the condensation of rhodamine B hydrazide with bis­(*o*-form­yl­phen­yl)diselenide. 
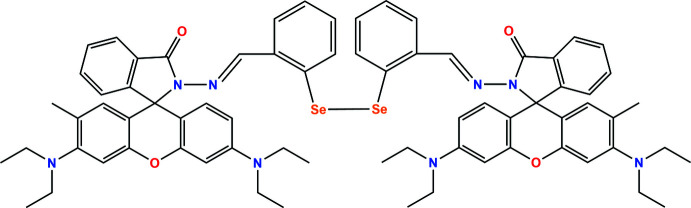


## Structural commentary

**Figure 1:**
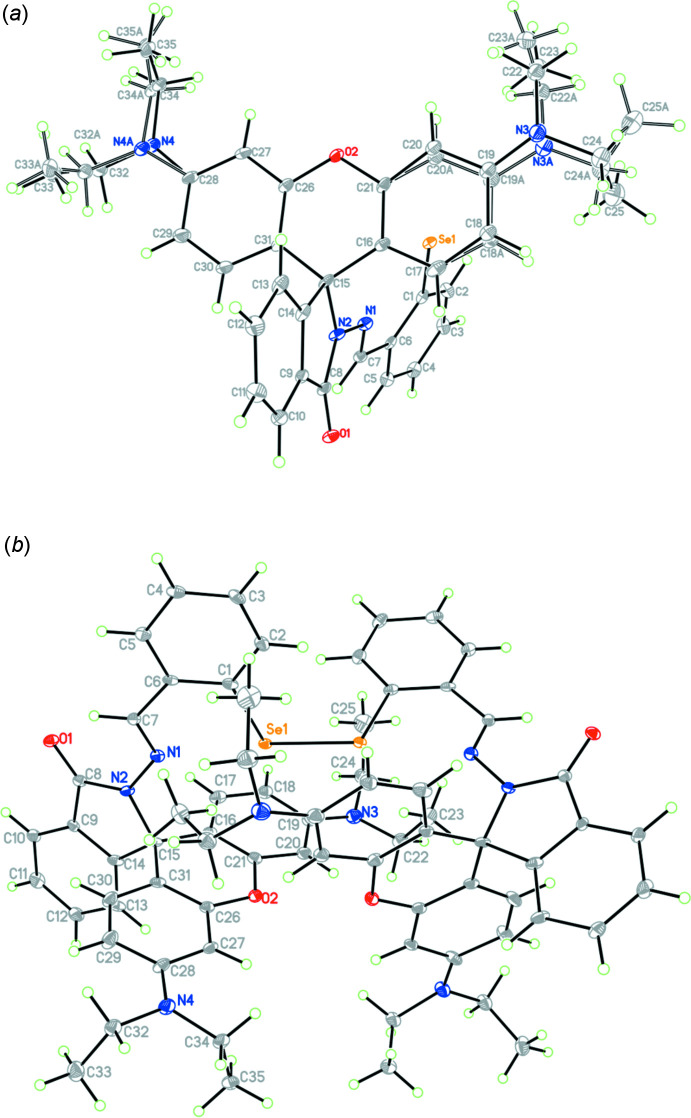
Diagram showing: (*a*) a half mol­ecule showing the disorder, (*b*) the major component of the title compound [symmetry operation: 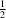 + *y*, − 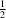 + *x*, 1 − *z*]. Displacement ellipsoids are shown at the 30% probability level.

The title compound (Fig. 1 ▸), a rare example of spiro bicyclic diselenide, crystallizes in the non-centrosymmetric polar tetra­gonal space group, *P* 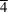 *b*2, as a racemic mixture. There is a half-mol­ecule in the asymmetric unit (*Z* = 4), and the structure was refined as an inversion twin [Flack parameter 0.05 (2); Parsons *et al.*, 2013 ▸]. The Se–Se unit is coplanar with both phenyl rings but the Se–aryl planes are essentially perpen­dic­ular to each other [C—Se—Se—C torsion angle of −88.9 (3)°]. The diethyl amine groups and their attached phenyl groups (C16–C21, N3/C18–C20/C22–C25 and C26–C31, N4/C32–C35) of the xanthene rings are disordered over two conformations with occupancies of 0.664 (19)/0.336 (19) and 0.665 (11)/0.335 (11), respectively. In both major and minor components, the diethyl amine nitro­gens are planar with the sum of the bond angles at N3/N3*A* being 358.5 and 359.5° and at N4/N4*A* being 357.5 and 357.4°, respectively. In order to investigate the pyramidal nature of the amine N atoms, the dihedral angles between the respective N—C_2_ groups and the attached phenyl rings were calculated and found to be 14.3 (7) and 14.8 (5) for N3 and N4, respectively. The Se—Se bond length of 2.3517 (17) Å and Se—C bond length of 1.939 (7) Å fall within the literature ranges of 2.287 to 3.051 Å and 1.91–1.97 Å, respectively (see CSD survey). The C—Se—Se—C torsion angle typically falls in the range of *ca* 73–128° (Dickson *et al.*, 1999 ▸). The observed C—Se—Se—C torsion angle, – 88.9 (3) °, results from the *syn* conformation around the Se–Se bridge. This conformation can be ration­al­ized in terms of repulsion of the 4*p* lone pairs at the Se centres. The dihedral angles between the mean planes of the central isoindoline (N2/C8/C9/C14/C15) and the phenyl rings (C1–C6 and C9–C14), are 26.8 (2) and 2.5 (4)°, respectively. The mean plane of the central xanthene ring (O2/C21/C16/C15/C31/C26) forms dihedral angles of 2.0 (5), 8.8 (9) and 1.7 (5), 7.9 (6)° with the peripheral phenyl rings (C16–C21, C16*A*–C21*A* and C26–C31, C26*A*–C31*A*, respectively). The isoindoline (N2/C8/C9/C14/C15) and xanthene (O2/C21/C16/C15/C31/C26) rings are essentially perpendicular to each other [dihedral angle of 89.8 (6)°].

## Supra­molecular features

**Figure 2:**
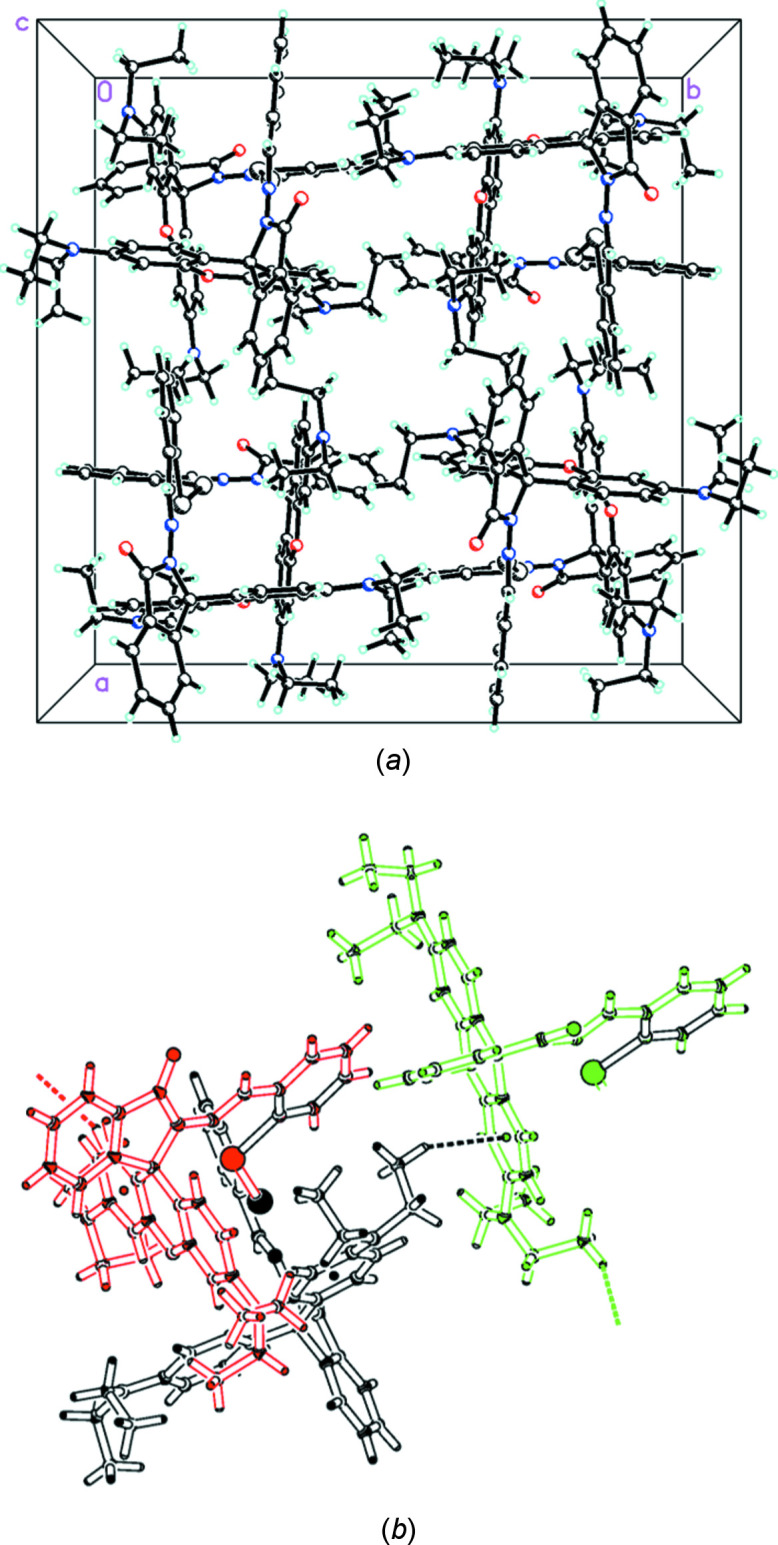
Packing diagram of (*a*) the title compound viewed along *c* axis and (*b*) partial packing showing the formation of C—H⋯π inter­actions (symmetry code: 1 − *y*, −1 + *x*, 1 − *z*).

The crystal packing of the title compound viewed along the *c* axis is presented in Fig. 2 ▸. The title compound packs in a way that allows close contacts between the oxygen atoms and hydrogen atoms of adjacent mol­ecules, leading to a network of C—H⋯O inter­actions involving donor atoms C7 (azomethine carbon) and C12 (aromatic carbon) with carbonyl oxygen O1 as acceptor with *D*⋯*A* distances of 3.391 (10) and 3.447 (10) Å, respectively (symmetry codes: *y* + 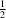 , *x* − 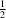 , −*z* + 2; 1 + *y*, 1 − *x*, 2 − *z*) between neighbouring mol­ecules (Table 1 ▸). An intra­molecular C—H⋯O hydrogen bond involving carbonyl oxygen, O1 and methine hydrogen, H7 with *D*⋯*A* distance of 2.940 (9) Å leading to an *S*(6) ring motif (Bernstein *et al.*, 1995 ▸) is also present. Furthermore, there exists a C—H⋯π inter­action between the H25*C* atom of the methyl carbon C25 and the centroid of the C16–C21 phenyl ring; symmetry code 1 − *y*, −1 + *x*, 1 − *z*. These inter­actions play a vital role in stabilizing the crystal packing within the crystal structure.

## Hirshfeld surface analysis

**Figure 3:**
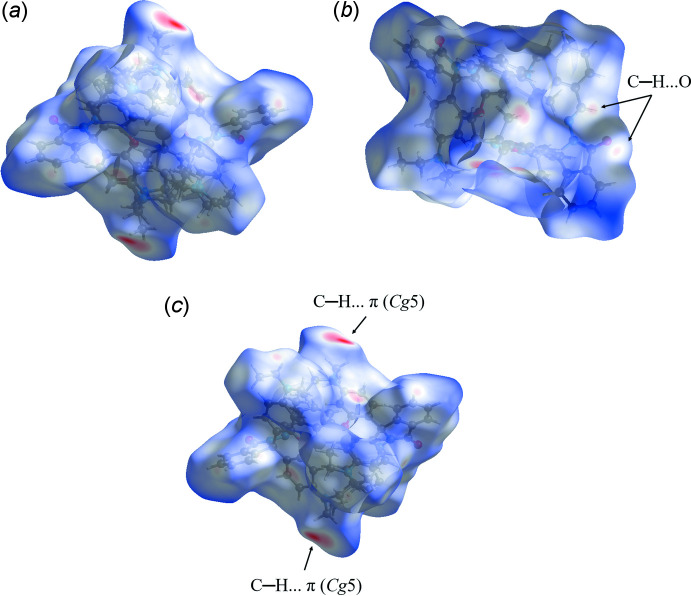
(*a*) A view of the three-dimensional Hirshfeld surface mapped over *d*_norm_ in the range −1.0432 to +2.0960 a.u. and views showing (*b*) C—H⋯O and (*c*) C—H⋯π inter­actions.

Hirshfeld surface (HS) calculations (Spackman & Jayatilaka, 2009 ▸) were performed on the title compound to further investigate the inter­molecular inter­actions. The Hirshfeld surface plotted over *d*_norm_ in the range −1.0432 to + 2.0960 a.u. generated using *CrystalExplorer 21.5* (Spackman *et al.*, 2021 ▸) is shown in Fig. 3 ▸. The red spots that appear around O1 are caused by the inter­molecular C7—H7⋯O1 and C12—H12⋯O1 inter­actions, which are important in the packing of the title mol­ecule. An intra­molecular C—H⋯O hydrogen bond is also indicated by the red spots near the hydrogen and oxygen atoms (Fig. 3 ▸ *b*). Bright-red spots on top and bottom of the HS near N3 indicate an inter­molecular C—H⋯π (ring) inter­action involving H25*B* of the C25 methyl group and a benzene ring (Fig. 3 ▸ *c*).

**Figure 4:**
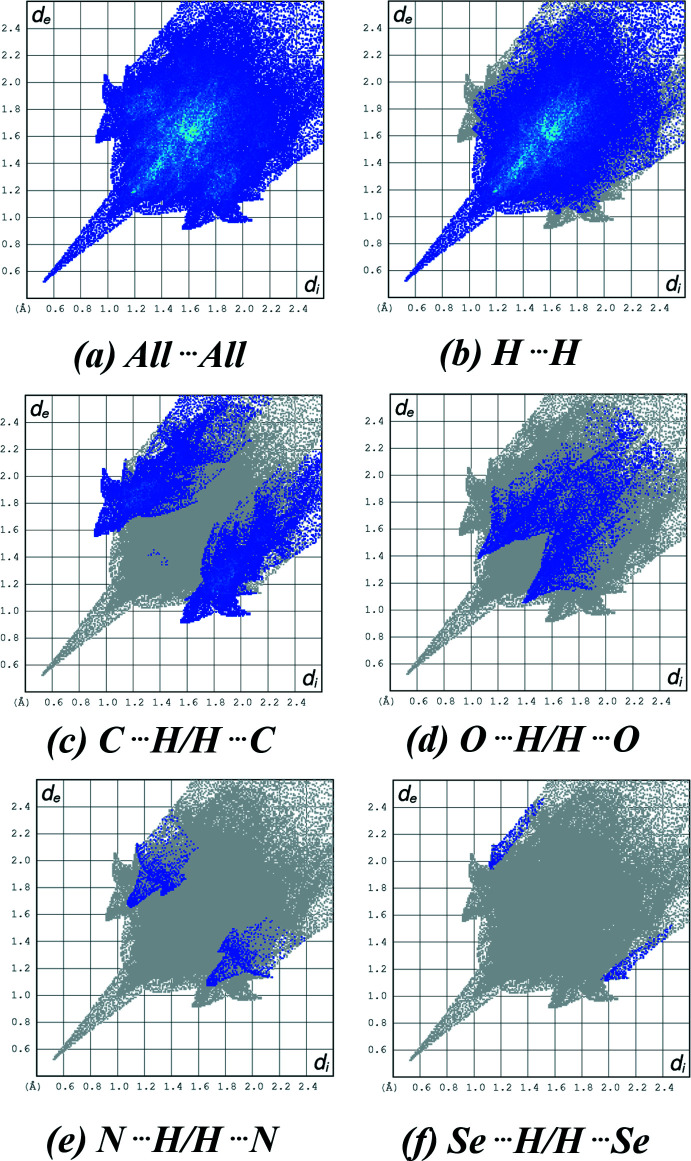
A view of the two-dimensional fingerprint plots for the title compound, showing (*a*) all inter­actions, and those delineated into (*b*) H⋯H (*c*) C⋯H/H⋯C (*d*) O⋯H/H⋯O (*e*) N⋯H/H⋯N and (*f*) Se⋯H/H⋯Se inter­actions. The *d*_i_ and *d*_e_ values are the closest inter­nal and external distances (in Å) from given points on the Hirshfeld surface.

The two-dimensional fingerprint plots (McKinnon *et al.*, 2007 ▸) were generated using *CrystalExplorer 21.5* encompassing all inter­molecular contacts, as well as the delineated specific contacts (Fig. 4 ▸). More significant contacts and their percentage contributions to the Hirshfeld surface are given in Table 2 ▸. The most important inter­action is H⋯H, contributing 68.1% to the overall crystal packing. The presence of C—H⋯π inter­actions is indicated by pairs of characteristic wings in the finger print plot representing C⋯H/H⋯C contacts with a 21.2% contribution to the HS. Pairs of scattered points of spikes are seen in the fingerprint plot delineated into O⋯H/H⋯O contacts (8.7% contribution to the HS). The lowest contributions are from N⋯H/H⋯N (1.6%) and Se⋯H/H⋯Se (0.4%) contacts. These inter­actions play a crucial role in the overall stabilization of the crystal packing.

## DFT Calculations

A density functional theory (DFT) geometry-optimized mol­ecular orbital calculation (*WebMOPro*; Polik & Schmidt, 2021 ▸) with the *GAUSSIAN 16* programme package (Frisch *et al.*, 2019 ▸) employing the B3LYP functional and 6-31 G(d) basis set (Becke, 1993 ▸) was performed on the title compound. Starting geometries were taken from the X-ray refinement data. Theoretical and experimental results related to bond lengths and angles are in good agreement (Table 3 ▸).

**Figure 5:**
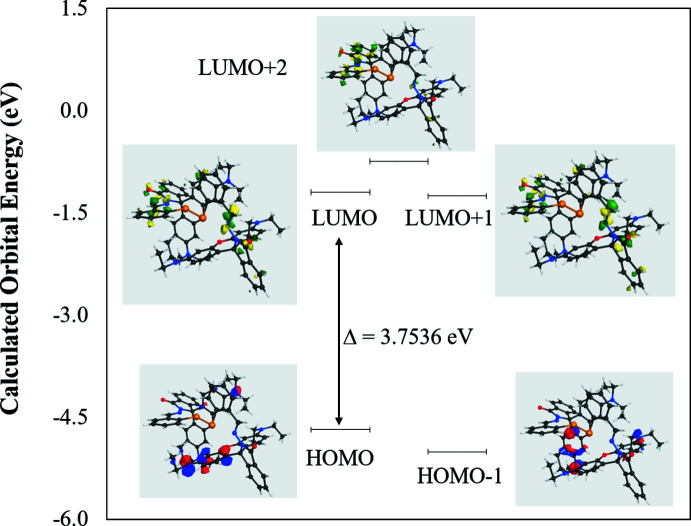
Calculated frontier mol­ecular orbitals of the title compound.

Calculated mol­ecular orbital energies (eV) for the surfaces of the frontier mol­ecular orbitals of the title compound are shown in Fig. 5 ▸. The HOMO (highest occupied mol­ecular orbital) acts as an electron donor and the LUMO (lowest unoccupied mol­ecular orbital) as an electron acceptor. Calculated numerical values for the title compound including, electronegativity (*c*), hardness (*h*), ionization enthalpy (*IE*), dipole moment (*m*), electron gain enthalpy (*EE*), electrophilicity (*ω*) and softness (*s*), are collated in Table 4 ▸. The significance of *h* and *s* is to evaluate both the reactivity and stability.

As shown in Fig. 5 ▸, the HOMO is mainly located on the xanthene phenyl ring and diethyl amine groups whereas the LUMO is distributed on the phenyl ring attached to selenium, azomethine and carbonyl group. In HOMO – 1, electron clouds are distributed on the azomethine group, the phenyl ring attached to selenium and the diethyl amine groups on the other side of the mol­ecule. In LUMO + 1, electron clouds are located on the isoindoline and azomethine groups of both sides of the mol­ecule whereas in LUMO + 2, it involves the selenium atom, phenyl ring, azomethine and isoindoline groups on one side of the mol­ecule. The energy band gap [Δ*E = E*_LUMO_ − *E*_HOMO_] of the mol­ecule is 3.7536 eV, and the frontier mol­ecular orbital energies, *E*_HOMO_ and *E*_LUMO_, are −5.0048 and −1.2512 eV, respectively.

**Figure 6:**
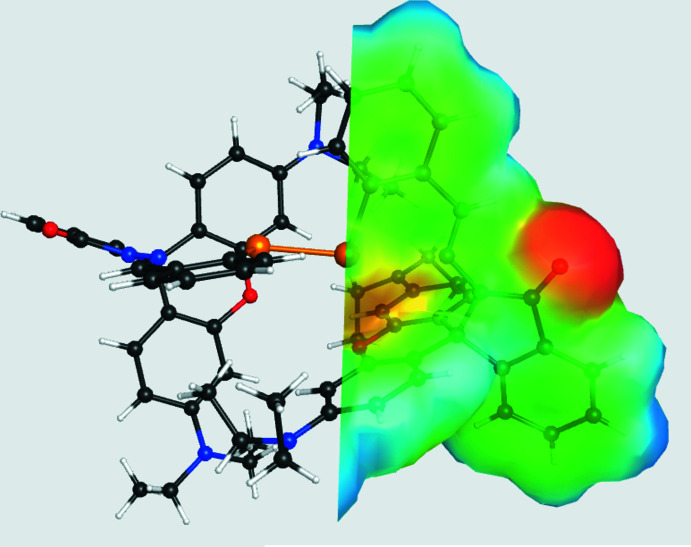
A view of the MEP plot of the title compound made using the 6–31 G(*d*) basis set at the B3LYP level of theory in the range − 0.0833 to 0.0321.

The mol­ecular electrostatic potential (MEP) map (Fig. 6 ▸) was calculated at the B3LYP/6–31G(d) level of theory. In the MEP diagram, the mol­ecular electrostatic potential is in the range −0.0833 to 0.0321 a.u. and the different electrostatic potentials at the surface of the mol­ecule are represented by different colours. Electrostatic potentials increase in the order red < yellow < green < blue, and red indicates the electron rich region and blue indicates the electron-deficient region. As shown in Fig. 6 ▸, the carbonyl groups are surrounded by negative charges, indicating some possible nucleophilic sites, whereas the positive charge regions are located on the H atoms indicating possible electrophilic sites.

## Database survey

A search of the Cambridge Structural Database (CSD, Version 5.42, update May 2021; Groom *et al.*, 2016 ▸) for the basic skeleton of this compound gave no hits. However, a CSD search on phen­yl–Se–Se–phenyl compounds gave 152 hits and 199 observations with the Se—Se distance ranging from 2.287 to 3.051 Å (with a mean value of 2.393 Å and a standard deviation 0.162). In the structures of CATWEB01, REDGAK, REDGEO and REDGUE (Panda *et al.*, 2012 ▸), the typical torsional angles of the selenium-attached phenyl ring (C—Se—Se—C) are *ca* 81° and those of CIDXET and CIDXUJ (Kulcsar *et al.*, 2007 ▸) are 80.9 and 114.0°, respectively.

## Synthesis and crystallization

**Figure 7:**
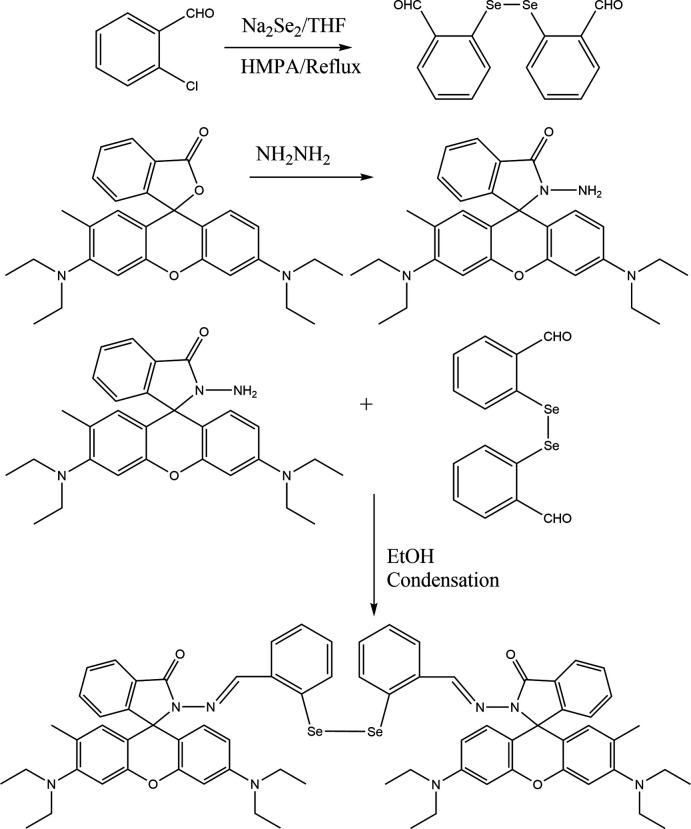
Scheme showing the reaction sequence for the synthesis of the title compound.

The title compound was obtained by the condensation of rhodamine B hydrazide (Leite *et al.*, 2013 ▸) and bis­(*o*-formyl­phen­yl)diselenide (Panda *et al.*, 2005 ▸) (see Fig. 7 ▸). In a typical experiment, a solution of rhodamine B hydrazide (0.228 g, 0.5 mmol) in ethanol (30 mL) was added dropwise to a solution of bis­(*o*-formyl­phen­yl)diselenide (0.184 g, 0.5 mmol) in ethanol (30 mL) over approximately 45 minutes in a dropping funnel. The solution mixture was stirred further for 4 h at room temperature. After cooling, the solid was filtered and washed three times with cold ethanol. Pale-yellow crystals of the title compound suitable for single-crystal X-ray diffraction study were obtained from chloro­form/pentane (1:1 mixture), yield 0.461 g, 81%, m.p. 519 K (Fig. 7 ▸). FT–IR (ATR): (*ν*, cm^−1^) = 3387, 2967, 1613, 1514, 1218, 1117, 753. ^1^H NMR [300 MHz, CDCl_3_, *δ* (ppm)]: 1.15 (24H, *t*, *J* = 7.2 Hz, NCH_2_CH_3_), 3.33 (16H, *q*, *J* = 7.2 Hz, NCH_2_CH_3_), 6.29 (4H, *s*, H-Ar), 6.43 (4H, *d*, *J* = 2.7 Hz, H-Ar), 6.46 (4H, *d*, *J* = 2.7 Hz, H-Ar), 7.09 (2H, *m*, H-Ar), 7.19 (4H, *m*, H-Ar), 7.43 (4H, *m*, H-Ar), 7.92 (4H, *m*, H-Ar), 8.60 (2H, *s*, N=C—H). ^13^C NMR [75 MHz, CDCl_3_, *δ* (ppm)]: 12.7 (NCH_2_CH_3_), 44.5 (NCH_2_CH_3_), 66.1 (spiro carbon), 98.2, 104.7, 108.2, 123.1, 123.9, 130.9, 132.6, 149.1, 151.7, 154.0, 166.3 (C=O).

## Refinement

Crystal data, data collection and structure refinement details are summarized in Table 5 ▸. All H atoms were positioned geometrically with C—H bond distances of 0.95 Å (aromatic H), 0.99 Å (methyl­ene H), 0.98 Å (methyl H) and were refined as riding with isotropic displacement parameters 1.2 and 1.5 times that of the adjacent carbon atoms. The title compound crystallized with disorder in the two diethyl amine groups attached to the xanthene ring. The disorder model included the phenyl rings to which these amine groups were attached. For these groups, the occupancy factors are 0.664 (19)/0.336 (19) and 0.665 (11)/0.335 (11). All atoms in the diethyl amine groups (N3/C18/C19/C20/C22/C23/C24/C25 and N4/C32/C33/C34/C35) were subject to displacement and positional restraints using SIMU and SAME instructions. For the SIMU command the esd’s used were 0.005 while for the SAME command the esd’s used were 0.003.

## Supplementary Material

Crystal structure: contains datablock(s) I. DOI: 10.1107/S2056989021013189/zl5020sup1.cif

Structure factors: contains datablock(s) I. DOI: 10.1107/S2056989021013189/zl5020Isup3.hkl

CCDC reference: 2127961

Additional supporting information:  crystallographic information; 3D view; checkCIF report

## Figures and Tables

**Table 1:** Hydrogen-bond geometry (Å, °)

*D*—H⋯*A*	*D*—H	H⋯*A*	*D*⋯*A*	*D*—H⋯*A*
C2—H2*A*⋯Se1^i^	0.95	2.82	3.436 (8)	124
C7—H7*A*⋯O1	0.95	2.43	2.940 (9)	114
C7—H7*A*⋯O1^ii^	0.95	2.63	3.391 (10)	137
C12—H12*A*⋯O1^iii^	0.95	2.56	3.447 (10)	156
C33*A*—H33*E*⋯Se1^i^	0.98	3.04	4.00 (3)	168

Symmetry codes: (i) y+{\script{1\over 2}}, x-{\script{1\over 2}}, -z+1; (ii) y+{\script{1\over 2}}, x-{\script{1\over 2}}, -z+2; (iii) y+1, -x+1, -z+2.

**Table 2:** Percentage contributions of inter­atomic contacts to the Hirshfeld surface for the title compound

Contact	Percentage contribution
H⋯H	68.1
C⋯H/H⋯C	21.2
O⋯H/H⋯O	8.7
N⋯H/H⋯N	1.6
Se⋯H/H⋯Se	0.4

**Table 3:** Comparison of selected (X-ray and DFT) bond lengths and angles (Å, °).

Bonds/Angles	X-ray	B3LYP/6–31G(*d*)
Se1—C1	1.939 (7)	1.941
Se1—Se1′	2.3517 (17)	2.356
O1—C8	1.222 (9)	1.224
O2—C21	1.315 (12)	1.374
O2—C26	1.349 (13)	1.368
N1—C7	1.280 (9)	1.291
N1—N2	1.380 (8)	1.353
N2—C8	1.378 (10)	1.392
N2—C15	1.507 (9)	1.513
C1—C6	1.415 (11)	1.42
C19—N3	1.426 (7)	1.383
C22—N3	1.446 (13)	1.464
C24—N3	1.466 (12)	1.461
N4—C28	1.423 (10)	1.39
N4—C32	1.543 (14)	1.461
N4—C34	1.481 (13)	1.462
C1—Se1—Se1′	103.5 (2)	102.711
C26—O2—C21	119.4 (9)	119.241
C7—N1—N2	121.3 (6)	122.983
N1—N2—C15	115.5 (5)	116.391
C2—C1—Se1	122.5 (6)	121.361
C6—C1—Se1	119.2 (5)	119.262
O1—C8—N2	125.8 (6)	126.543
C8—N2—C15	115.3 (5)	114.337
O1—C8—C9	129.0 (7)	128.268
N2—C8—C9	105.2 (6)	105.19
C19—N3—C22	120.9 (7)	120.635
C19—N3—C24	119.4 (7)	120.99
C22—N3—C24	118.2 (7)	118.187
C28—N4—C32	120.2 (7)	120.875
C28—N4—C34	120.1 (8)	120.772
C32—N4—C34	117.2 (9)	117.917
C1—Se1—Se1′—C1′	−88.9 (6)	−73.195

**Table 4:** Calculated energies

Property	
Total energy *TE* (eV)	−224397
*E*_HOMO_	−5.0048
*E*_LUMO_	−1.2512
Gap, Δ*E* (eV)	3.7536
Dipole moment, μ (Debye)	7.182
Ionization enthalpy, *IE* (eV)	5.0048
Electron gain enthalpy, *EE* (eV)	1.2512
Electronegativity, χ	3.128
Hardness, η	1.8768
Softness, σ	0.5328
Electrophilicity index, ω	2.6066

**Table 5:** Experimental details

Crystal data	
Chemical formula	C_70_H_70_N_8_O_4_Se_2_
*M*_r_	1245.26
Crystal system, space group	Tetragonal, *P*\overline{4}*b*2
Temperature (K)	100
*a*, *c* (Å)	21.507 (4), 13.434 (4)
*V* (Å^3^)	6214 (3)
*Z*	4
Radiation type	Mo *K*α
μ (mm^−1^)	1.25
Crystal size (mm)	0.27 × 0.23 × 0.08

Data collection	
Diffractometer	Bruker APEXII CCD
Absorption correction	Multi-scan (*SADABS*; Krause *et al.*, 2015 ▸)
*T*_min_, *T*_max_	0.566, 0.746
No. of measured, independent and observed [*I* > 2σ(*I*)] reflections	7696, 7696, 6090
*R*_int_	0.119
(sin θ/λ)_max_ (Å^−1^)	0.666

Refinement	
*R*[*F* ^2^ > 2σ(*F* ^2^)], *wR*(*F* ^2^), *S*	0.070, 0.142, 1.08
No. of reflections	7696
No. of parameters	540
No. of restraints	696
H-atom treatment	H-atom parameters constrained
Δρ_max_, Δρ_min_ (e Å^−3^)	0.52, −0.97
Absolute structure	Refined as an inversion twin
Absolute structure parameter	0.05 (2)

Computer programs: *APEX2* and *SAINT* (Bruker 2005 ▸), *SHELXT* (Sheldrick, 2015*a* ▸), *SHELXL2018/3* (Sheldrick, 2015*b* ▸), *SHELXTL* (Sheldrick, 2008 ▸) and *publCIF* (Westrip (2010 ▸).

## supplementary crystallographic information

### Crystal data

**Table:**

C_70_H_70_N_8_O_4_Se_2_	*D*_x_ = 1.331 Mg m^−^^3^
*M**_r_* = 1245.26	Mo *K*α radiation, λ = 0.71073 Å
Tetragonal, *P*4*b*2	Cell parameters from 7629 reflections
*a* = 21.507 (4) Å	θ = 2.4–30.1°
*c* = 13.434 (4) Å	µ = 1.25 mm^−^^1^
*V* = 6214 (3) Å^3^	*T* = 100 K
*Z* = 4	Plate, pale yellow
*F*(000) = 2584	0.27 × 0.23 × 0.08 mm

### Data collection

**Table:**

Bruker APEXII CCD diffractometer	6090 reflections with *I* > 2σ(*I*)
φ and ω scans	*R*_int_ = 0.119
Absorption correction: multi-scan (SADABS; Krause *et al.*, 2015)	θ_max_ = 28.3°, θ_min_ = 2.0°
*T*_min_ = 0.566, *T*_max_ = 0.746	*h* = −24→25
7696 measured reflections	*k* = −25→25
7696 independent reflections	*l* = −16→16

### Refinement

**Table:**

Refinement on *F*^2^	Secondary atom site location: difference Fourier map
Least-squares matrix: full	Hydrogen site location: inferred from neighbouring sites
*R*[*F*^2^ > 2σ(*F*^2^)] = 0.070	H-atom parameters constrained
*wR*(*F*^2^) = 0.142	*w* = 1/[σ^2^(*F*_o_^2^) + (0.040*P*)^2^ + 13.6633*P*] where *P* = (*F*_o_^2^ + 2*F*_c_^2^)/3
*S* = 1.08	(Δ/σ)_max_ = 0.001
7696 reflections	Δρ_max_ = 0.52 e Å^−^^3^
540 parameters	Δρ_min_ = −0.96 e Å^−^^3^
696 restraints	Absolute structure: Refined as an inversion twin
Primary atom site location: dual	Absolute structure parameter: 0.05 (2)

### Special details

**Table:**

Geometry. All esds (except the esd in the dihedral angle between two l.s. planes) are estimated using the full covariance matrix. The cell esds are taken into account individually in the estimation of esds in distances, angles and torsion angles; correlations between esds in cell parameters are only used when they are defined by crystal symmetry. An approximate (isotropic) treatment of cell esds is used for estimating esds involving l.s. planes.
Refinement. Refined as a 2-component inversion twin.

### Fractional atomic coordinates and isotropic or equivalent isotropic displacement parameters (Å^2^)

**Table:**

	*x*	*y*	*z*	*U*_iso_*/*U*_eq_	Occ. (<1)
Se1	0.70568 (3)	0.18183 (3)	0.58326 (6)	0.01973 (16)	
O1	0.7532 (2)	0.1249 (2)	0.9772 (4)	0.0257 (12)	
O2	0.8536 (3)	0.2775 (2)	0.6006 (4)	0.0307 (13)	
N1	0.7287 (3)	0.1778 (3)	0.7778 (5)	0.0218 (13)	
N2	0.7820 (3)	0.1786 (3)	0.8351 (5)	0.0215 (13)	
C1	0.6247 (3)	0.1743 (3)	0.6457 (6)	0.0209 (15)	
C2	0.5709 (3)	0.1704 (3)	0.5920 (7)	0.0269 (17)	
H2A	0.573364	0.172617	0.521440	0.032*	
C3	0.5136 (4)	0.1636 (4)	0.6348 (6)	0.0305 (19)	
H3A	0.477457	0.160360	0.594590	0.037*	
C4	0.5089 (3)	0.1615 (4)	0.7383 (7)	0.0303 (19)	
H4A	0.469386	0.156737	0.768954	0.036*	
C5	0.5619 (3)	0.1662 (4)	0.7962 (6)	0.0253 (17)	
H5A	0.558725	0.165049	0.866677	0.030*	
C6	0.6207 (3)	0.1729 (3)	0.7508 (6)	0.0215 (16)	
C7	0.6747 (3)	0.1760 (3)	0.8178 (6)	0.0222 (16)	
H7A	0.669701	0.176542	0.888011	0.027*	
C8	0.7925 (3)	0.1505 (3)	0.9257 (6)	0.0232 (15)	
C9	0.8604 (3)	0.1565 (3)	0.9431 (5)	0.0228 (17)	
C10	0.8957 (4)	0.1375 (4)	1.0242 (6)	0.0306 (19)	
H10A	0.877362	0.115811	1.078296	0.037*	
C11	0.9584 (4)	0.1513 (4)	1.0233 (7)	0.037 (2)	
H11A	0.983691	0.137835	1.077174	0.044*	
C12	0.9853 (4)	0.1842 (4)	0.9463 (6)	0.037 (2)	
H12A	1.028150	0.194827	0.949810	0.045*	
C13	0.9506 (4)	0.2021 (4)	0.8633 (7)	0.0317 (19)	
H13A	0.969281	0.223137	0.808773	0.038*	
C14	0.8875 (3)	0.1879 (3)	0.8636 (6)	0.0251 (17)	
C15	0.8387 (3)	0.2057 (3)	0.7855 (6)	0.0221 (16)	
C16	0.8320 (7)	0.2769 (4)	0.7760 (12)	0.0246 (13)	0.664 (19)
C17	0.8171 (5)	0.3120 (4)	0.8595 (9)	0.0261 (14)	0.664 (19)
H17A	0.810023	0.292093	0.921568	0.031*	0.664 (19)
C18	0.8125 (5)	0.3763 (4)	0.8522 (7)	0.0276 (14)	0.664 (19)
H18A	0.802296	0.400347	0.909233	0.033*	0.664 (19)
C19	0.8228 (6)	0.4055 (4)	0.7613 (7)	0.0267 (12)	0.664 (19)
C20	0.8377 (8)	0.3704 (5)	0.6778 (8)	0.0253 (12)	0.664 (19)
H20A	0.844811	0.390334	0.615758	0.030*	0.664 (19)
C21	0.8423 (9)	0.3061 (5)	0.6852 (10)	0.0245 (12)	0.664 (19)
N3	0.8171 (5)	0.4714 (4)	0.7532 (8)	0.0295 (13)	0.664 (19)
C22	0.8049 (7)	0.5092 (5)	0.8399 (10)	0.0306 (16)	0.664 (19)
H22A	0.772449	0.488677	0.880586	0.037*	0.664 (19)
H22B	0.788165	0.549841	0.817849	0.037*	0.664 (19)
C23	0.8617 (8)	0.5205 (6)	0.9047 (11)	0.037 (3)	0.664 (19)
H23A	0.852407	0.553040	0.953573	0.056*	0.664 (19)
H23B	0.896586	0.533822	0.862790	0.056*	0.664 (19)
H23C	0.872832	0.482036	0.939413	0.056*	0.664 (19)
C24	0.8381 (7)	0.5024 (6)	0.6620 (9)	0.0298 (15)	0.664 (19)
H24A	0.822744	0.545818	0.662541	0.036*	0.664 (19)
H24B	0.819286	0.481215	0.604023	0.036*	0.664 (19)
C25	0.9087 (8)	0.5033 (12)	0.6489 (16)	0.031 (3)	0.664 (19)
H25A	0.919134	0.524192	0.586220	0.047*	0.664 (19)
H25B	0.924392	0.460560	0.647392	0.047*	0.664 (19)
H25C	0.927740	0.525770	0.704517	0.047*	0.664 (19)
C16A	0.8351 (14)	0.2773 (7)	0.780 (2)	0.0248 (14)	0.336 (19)
C17A	0.8317 (11)	0.3109 (8)	0.8683 (19)	0.0261 (15)	0.336 (19)
H17B	0.830645	0.289786	0.930371	0.031*	0.336 (19)
C18A	0.8300 (11)	0.3755 (8)	0.8657 (14)	0.0266 (15)	0.336 (19)
H18B	0.827661	0.398511	0.925919	0.032*	0.336 (19)
C19A	0.8316 (11)	0.4064 (7)	0.7749 (13)	0.0270 (12)	0.336 (19)
C20A	0.8350 (16)	0.3728 (9)	0.6867 (14)	0.0254 (13)	0.336 (19)
H20B	0.836078	0.393930	0.624620	0.030*	0.336 (19)
C21A	0.8367 (18)	0.3082 (9)	0.689 (2)	0.0246 (13)	0.336 (19)
N3A	0.8297 (10)	0.4726 (8)	0.7720 (13)	0.0291 (13)	0.336 (19)
C22A	0.8308 (12)	0.5089 (9)	0.8626 (17)	0.0309 (17)	0.336 (19)
H22C	0.800367	0.491063	0.910067	0.037*	0.336 (19)
H22D	0.817367	0.551850	0.847216	0.037*	0.336 (19)
C23A	0.8943 (14)	0.5112 (12)	0.9123 (19)	0.038 (3)	0.336 (19)
H23D	0.889360	0.523101	0.982309	0.057*	0.336 (19)
H23E	0.920456	0.541792	0.878306	0.057*	0.336 (19)
H23F	0.913845	0.470107	0.908442	0.057*	0.336 (19)
C24A	0.8356 (12)	0.5051 (11)	0.6767 (15)	0.0298 (16)	0.336 (19)
H24C	0.819079	0.547850	0.684418	0.036*	0.336 (19)
H24D	0.809503	0.483585	0.626721	0.036*	0.336 (19)
C25A	0.9021 (15)	0.509 (3)	0.637 (3)	0.031 (3)	0.336 (19)
H25D	0.901289	0.520804	0.566831	0.047*	0.336 (19)
H25E	0.922335	0.468488	0.644552	0.047*	0.336 (19)
H25F	0.925212	0.540334	0.675210	0.047*	0.336 (19)
C26	0.8598 (9)	0.2150 (6)	0.5998 (10)	0.0279 (16)	0.665 (11)
C27	0.8741 (5)	0.1898 (4)	0.5073 (8)	0.0299 (16)	0.665 (11)
H27A	0.880123	0.216328	0.451607	0.036*	0.665 (11)
C28	0.8797 (5)	0.1258 (4)	0.4964 (6)	0.0323 (14)	0.665 (11)
C29	0.8709 (5)	0.0870 (5)	0.5780 (7)	0.0310 (16)	0.665 (11)
H29A	0.874735	0.043210	0.570492	0.037*	0.665 (11)
C30	0.8566 (9)	0.1122 (8)	0.6704 (7)	0.0298 (15)	0.665 (11)
H30A	0.850618	0.085668	0.726146	0.036*	0.665 (11)
C31	0.8510 (12)	0.1762 (8)	0.6813 (8)	0.0272 (14)	0.665 (11)
N4	0.8992 (5)	0.1018 (4)	0.4027 (9)	0.0376 (14)	0.665 (11)
C32	0.9076 (6)	0.1459 (6)	0.3131 (10)	0.0414 (18)	0.665 (11)
H32A	0.931083	0.183592	0.332541	0.050*	0.665 (11)
H32B	0.929969	0.124816	0.258215	0.050*	0.665 (11)
C33	0.8448 (7)	0.1617 (8)	0.2835 (13)	0.049 (3)	0.665 (11)
H33A	0.846143	0.191657	0.228587	0.074*	0.665 (11)
H33B	0.822739	0.180067	0.340131	0.074*	0.665 (11)
H33C	0.823053	0.124025	0.261767	0.074*	0.665 (11)
C34	0.8932 (6)	0.0346 (5)	0.3811 (11)	0.0411 (18)	0.665 (11)
H34A	0.915231	0.009836	0.432176	0.049*	0.665 (11)
H34B	0.911327	0.024895	0.315153	0.049*	0.665 (11)
C35	0.8198 (7)	0.0183 (7)	0.3820 (13)	0.054 (3)	0.665 (11)
H35A	0.814274	−0.026890	0.382246	0.081*	0.665 (11)
H35B	0.800074	0.035921	0.322558	0.081*	0.665 (11)
H35C	0.800606	0.036133	0.441678	0.081*	0.665 (11)
C26A	0.8526 (18)	0.2148 (12)	0.5986 (19)	0.0283 (16)	0.335 (11)
C27A	0.8569 (11)	0.1885 (8)	0.5044 (16)	0.0305 (16)	0.335 (11)
H27B	0.859045	0.214396	0.447248	0.037*	0.335 (11)
C28A	0.8579 (10)	0.1242 (8)	0.4938 (12)	0.0332 (15)	0.335 (11)
C29A	0.8547 (11)	0.0862 (11)	0.5774 (13)	0.0312 (16)	0.335 (11)
H29B	0.855455	0.042316	0.570102	0.037*	0.335 (11)
C30A	0.8504 (19)	0.1126 (15)	0.6716 (13)	0.0296 (16)	0.335 (11)
H30B	0.848250	0.086658	0.728742	0.036*	0.335 (11)
C31A	0.849 (2)	0.1769 (16)	0.6822 (15)	0.0278 (15)	0.335 (11)
N4A	0.8663 (9)	0.0987 (8)	0.3969 (14)	0.0386 (15)	0.335 (11)
C32A	0.8874 (11)	0.1405 (10)	0.3100 (16)	0.0406 (19)	0.335 (11)
H32C	0.907631	0.178796	0.335110	0.049*	0.335 (11)
H32D	0.916908	0.118233	0.266124	0.049*	0.335 (11)
C33A	0.8305 (13)	0.1553 (15)	0.257 (2)	0.045 (3)	0.335 (11)
H33D	0.837508	0.191426	0.214184	0.067*	0.335 (11)
H33E	0.797618	0.164806	0.305268	0.067*	0.335 (11)
H33F	0.818059	0.119616	0.216466	0.067*	0.335 (11)
C34A	0.8740 (11)	0.0307 (8)	0.3843 (19)	0.0410 (19)	0.335 (11)
H34C	0.840464	0.013913	0.341508	0.049*	0.335 (11)
H34D	0.871955	0.009659	0.449758	0.049*	0.335 (11)
C35A	0.9409 (11)	0.0191 (12)	0.333 (2)	0.051 (4)	0.335 (11)
H35D	0.937979	−0.015795	0.286465	0.076*	0.335 (11)
H35E	0.971630	0.009329	0.384804	0.076*	0.335 (11)
H35F	0.953808	0.056594	0.297335	0.076*	0.335 (11)

### Atomic displacement parameters (Å^2^)

**Table:**

	*U*^11^	*U*^22^	*U*^33^	*U*^12^	*U*^13^	*U*^23^
Se1	0.0141 (3)	0.0190 (3)	0.0260 (3)	−0.0019 (3)	−0.0068 (3)	0.0070 (3)
O1	0.023 (3)	0.022 (3)	0.031 (3)	−0.001 (2)	−0.005 (2)	0.010 (2)
O2	0.046 (3)	0.019 (3)	0.027 (3)	−0.009 (2)	−0.002 (3)	0.006 (2)
N1	0.017 (3)	0.022 (3)	0.027 (3)	0.000 (2)	−0.008 (2)	0.007 (3)
N2	0.018 (3)	0.020 (3)	0.027 (3)	−0.003 (2)	−0.007 (2)	0.012 (3)
C1	0.017 (3)	0.013 (3)	0.033 (4)	0.003 (3)	−0.006 (3)	0.002 (3)
C2	0.017 (3)	0.037 (4)	0.027 (4)	0.004 (3)	−0.003 (3)	−0.003 (4)
C3	0.019 (4)	0.037 (5)	0.036 (5)	0.005 (3)	−0.008 (3)	−0.006 (4)
C4	0.012 (3)	0.036 (5)	0.043 (5)	0.000 (3)	0.001 (4)	−0.004 (4)
C5	0.016 (4)	0.029 (4)	0.031 (4)	0.005 (3)	−0.002 (3)	−0.002 (3)
C6	0.019 (3)	0.013 (3)	0.032 (4)	0.000 (3)	−0.002 (3)	0.002 (3)
C7	0.022 (4)	0.018 (3)	0.026 (4)	0.000 (3)	−0.003 (3)	0.004 (3)
C8	0.026 (3)	0.012 (3)	0.032 (4)	−0.002 (2)	−0.009 (4)	0.005 (3)
C9	0.027 (4)	0.019 (3)	0.022 (4)	−0.001 (3)	−0.011 (3)	0.003 (3)
C10	0.037 (5)	0.028 (4)	0.027 (4)	−0.003 (3)	−0.016 (4)	0.006 (4)
C11	0.033 (5)	0.041 (5)	0.036 (5)	0.007 (4)	−0.018 (4)	0.005 (4)
C12	0.028 (4)	0.046 (5)	0.038 (5)	−0.004 (4)	−0.014 (4)	0.003 (4)
C13	0.025 (4)	0.024 (4)	0.046 (5)	−0.003 (3)	−0.012 (4)	0.001 (4)
C14	0.021 (4)	0.018 (4)	0.036 (5)	−0.003 (3)	−0.009 (3)	0.003 (3)
C15	0.015 (3)	0.014 (3)	0.037 (4)	−0.002 (3)	−0.010 (3)	0.011 (3)
C16	0.022 (3)	0.020 (2)	0.032 (3)	−0.002 (2)	−0.004 (2)	0.008 (2)
C17	0.025 (3)	0.022 (2)	0.032 (3)	−0.001 (2)	−0.003 (3)	0.008 (2)
C18	0.028 (3)	0.023 (2)	0.033 (3)	0.000 (2)	−0.002 (3)	0.006 (2)
C19	0.027 (2)	0.021 (2)	0.032 (3)	0.000 (2)	−0.001 (2)	0.007 (2)
C20	0.025 (3)	0.020 (2)	0.031 (3)	−0.002 (2)	−0.003 (2)	0.009 (2)
C21	0.022 (3)	0.020 (2)	0.031 (3)	−0.002 (2)	−0.004 (2)	0.008 (2)
N3	0.033 (3)	0.021 (2)	0.034 (3)	0.000 (2)	−0.001 (2)	0.005 (2)
C22	0.034 (3)	0.023 (3)	0.035 (3)	0.000 (3)	0.000 (3)	0.005 (3)
C23	0.043 (5)	0.033 (4)	0.036 (5)	0.004 (4)	−0.006 (5)	0.005 (4)
C24	0.035 (3)	0.020 (3)	0.035 (3)	0.000 (2)	−0.001 (3)	0.007 (3)
C25	0.038 (5)	0.019 (5)	0.037 (5)	−0.003 (4)	−0.005 (4)	0.005 (4)
C16A	0.022 (3)	0.020 (2)	0.032 (3)	−0.001 (2)	−0.004 (2)	0.008 (2)
C17A	0.024 (3)	0.022 (2)	0.032 (3)	−0.001 (3)	−0.003 (3)	0.007 (2)
C18A	0.026 (3)	0.022 (2)	0.032 (3)	0.000 (3)	−0.003 (3)	0.007 (2)
C19A	0.028 (3)	0.021 (2)	0.032 (3)	−0.001 (2)	−0.001 (2)	0.007 (2)
C20A	0.025 (3)	0.020 (2)	0.031 (3)	−0.002 (2)	−0.003 (2)	0.008 (2)
C21A	0.023 (3)	0.020 (2)	0.031 (3)	−0.001 (2)	−0.004 (2)	0.008 (2)
N3A	0.033 (3)	0.021 (2)	0.034 (3)	0.000 (2)	−0.001 (2)	0.006 (2)
C22A	0.035 (3)	0.023 (3)	0.034 (3)	0.000 (3)	−0.001 (3)	0.005 (3)
C23A	0.043 (7)	0.032 (6)	0.039 (6)	−0.006 (6)	−0.004 (7)	0.000 (6)
C24A	0.035 (3)	0.020 (3)	0.035 (3)	0.000 (3)	−0.001 (3)	0.006 (3)
C25A	0.039 (5)	0.019 (5)	0.036 (5)	−0.001 (5)	−0.005 (5)	0.006 (5)
C26	0.026 (4)	0.021 (2)	0.037 (3)	−0.001 (3)	−0.009 (3)	0.004 (2)
C27	0.027 (4)	0.024 (2)	0.039 (3)	−0.002 (3)	−0.010 (3)	0.004 (2)
C28	0.029 (3)	0.026 (2)	0.042 (2)	−0.001 (3)	−0.009 (3)	0.002 (2)
C29	0.028 (4)	0.025 (2)	0.040 (3)	−0.001 (3)	−0.008 (3)	0.004 (2)
C30	0.027 (4)	0.024 (2)	0.039 (3)	−0.001 (3)	−0.008 (3)	0.005 (2)
C31	0.024 (3)	0.021 (2)	0.037 (3)	−0.002 (2)	−0.009 (2)	0.005 (2)
N4	0.033 (3)	0.033 (2)	0.047 (3)	−0.001 (3)	−0.007 (3)	−0.003 (2)
C32	0.036 (4)	0.038 (3)	0.050 (3)	0.001 (3)	−0.008 (3)	−0.007 (3)
C33	0.041 (6)	0.051 (5)	0.056 (5)	−0.004 (5)	−0.005 (5)	−0.015 (5)
C34	0.038 (4)	0.036 (3)	0.050 (3)	−0.001 (3)	−0.008 (3)	−0.002 (3)
C35	0.050 (6)	0.050 (5)	0.063 (6)	0.003 (5)	−0.009 (5)	−0.003 (5)
C26A	0.026 (4)	0.022 (3)	0.038 (3)	−0.002 (3)	−0.009 (3)	0.004 (2)
C27A	0.028 (4)	0.024 (3)	0.039 (3)	−0.002 (3)	−0.009 (3)	0.003 (2)
C28A	0.030 (4)	0.027 (2)	0.042 (2)	−0.001 (3)	−0.009 (3)	0.002 (2)
C29A	0.028 (4)	0.025 (2)	0.040 (3)	−0.001 (3)	−0.008 (3)	0.004 (2)
C30A	0.026 (4)	0.024 (2)	0.039 (3)	−0.001 (3)	−0.008 (3)	0.005 (2)
C31A	0.025 (4)	0.022 (2)	0.037 (3)	−0.002 (3)	−0.009 (3)	0.005 (2)
N4A	0.035 (4)	0.034 (2)	0.047 (3)	−0.001 (3)	−0.008 (3)	−0.003 (2)
C32A	0.036 (4)	0.037 (3)	0.049 (3)	−0.001 (3)	−0.008 (4)	−0.006 (3)
C33A	0.038 (6)	0.045 (5)	0.051 (6)	0.000 (5)	−0.007 (6)	−0.010 (5)
C34A	0.037 (4)	0.035 (3)	0.050 (3)	−0.001 (3)	−0.008 (4)	−0.003 (3)
C35A	0.049 (7)	0.040 (6)	0.063 (7)	−0.002 (6)	−0.006 (6)	−0.002 (6)

### Geometric parameters (Å, º)

**Table:**

Se1—C1	1.939 (7)	C18A—H18B	0.9500
Se1—Se1^i^	2.3517 (17)	C19A—C20A	1.3900
O1—C8	1.222 (9)	C19A—N3A	1.425 (8)
O2—C21	1.315 (12)	C20A—C21A	1.3900
O2—C26A	1.35 (3)	C20A—H20B	0.9500
O2—C26	1.349 (13)	N3A—C22A	1.446 (13)
O2—C21A	1.41 (2)	N3A—C24A	1.465 (12)
N1—C7	1.280 (9)	C22A—C23A	1.521 (17)
N1—N2	1.380 (8)	C22A—H22C	0.9900
N2—C8	1.378 (10)	C22A—H22D	0.9900
N2—C15	1.507 (9)	C23A—H23D	0.9800
C1—C2	1.367 (10)	C23A—H23E	0.9800
C1—C6	1.415 (11)	C23A—H23F	0.9800
C2—C3	1.368 (11)	C24A—C25A	1.527 (12)
C2—H2A	0.9500	C24A—H24C	0.9900
C3—C4	1.394 (12)	C24A—H24D	0.9900
C3—H3A	0.9500	C25A—H25D	0.9800
C4—C5	1.384 (11)	C25A—H25E	0.9800
C4—H4A	0.9500	C25A—H25F	0.9800
C5—C6	1.411 (10)	C26—C27	1.3900
C5—H5A	0.9500	C26—C31	1.3900
C6—C7	1.471 (10)	C27—C28	1.3900
C7—H7A	0.9500	C27—H27A	0.9500
C8—C9	1.484 (10)	C28—C29	1.3900
C9—C10	1.389 (10)	C28—N4	1.423 (10)
C9—C14	1.392 (11)	C29—C30	1.3900
C10—C11	1.381 (12)	C29—H29A	0.9500
C10—H10A	0.9500	C30—C31	1.3900
C11—C12	1.381 (12)	C30—H30A	0.9500
C11—H11A	0.9500	N4—C34	1.481 (13)
C12—C13	1.397 (11)	N4—C32	1.543 (14)
C12—H12A	0.9500	C32—C33	1.448 (16)
C13—C14	1.391 (11)	C32—H32A	0.9900
C13—H13A	0.9500	C32—H32B	0.9900
C14—C15	1.534 (10)	C33—H33A	0.9800
C15—C31A	1.537 (18)	C33—H33B	0.9800
C15—C16A	1.542 (17)	C33—H33C	0.9800
C15—C16	1.543 (10)	C34—C35	1.616 (19)
C15—C31	1.559 (11)	C34—H34A	0.9900
C16—C17	1.3900	C34—H34B	0.9900
C16—C21	1.3900	C35—H35A	0.9800
C17—C18	1.3900	C35—H35B	0.9800
C17—H17A	0.9500	C35—H35C	0.9800
C18—C19	1.3900	C26A—C27A	1.3900
C18—H18A	0.9500	C26A—C31A	1.3900
C19—C20	1.3900	C27A—C28A	1.3900
C19—N3	1.426 (7)	C27A—H27B	0.9500
C20—C21	1.3900	C28A—C29A	1.3900
C20—H20A	0.9500	C28A—N4A	1.424 (11)
N3—C22	1.446 (13)	C29A—C30A	1.3900
N3—C24	1.466 (12)	C29A—H29B	0.9500
C22—C23	1.520 (17)	C30A—C31A	1.3900
C22—H22A	0.9900	C30A—H30B	0.9500
C22—H22B	0.9900	N4A—C34A	1.481 (13)
C23—H23A	0.9800	N4A—C32A	1.543 (15)
C23—H23B	0.9800	C32A—C33A	1.448 (17)
C23—H23C	0.9800	C32A—H32C	0.9900
C24—C25	1.528 (12)	C32A—H32D	0.9900
C24—H24A	0.9900	C33A—H33D	0.9800
C24—H24B	0.9900	C33A—H33E	0.9800
C25—H25A	0.9800	C33A—H33F	0.9800
C25—H25B	0.9800	C34A—C35A	1.615 (19)
C25—H25C	0.9800	C34A—H34C	0.9900
C16A—C17A	1.3900	C34A—H34D	0.9900
C16A—C21A	1.3900	C35A—H35D	0.9800
C17A—C18A	1.3900	C35A—H35E	0.9800
C17A—H17B	0.9500	C35A—H35F	0.9800
C18A—C19A	1.3900		
			
C1—Se1—Se1^i^	103.5 (2)	C20A—C21A—C16A	120.0
C21—O2—C26	119.4 (9)	C20A—C21A—O2	117.0 (15)
C26A—O2—C21A	118.8 (16)	C16A—C21A—O2	121.6 (15)
C7—N1—N2	121.3 (6)	C19A—N3A—C22A	121.1 (8)
C8—N2—N1	128.5 (6)	C19A—N3A—C24A	119.9 (8)
C8—N2—C15	115.3 (5)	C22A—N3A—C24A	118.5 (8)
N1—N2—C15	115.5 (5)	N3A—C22A—C23A	113.7 (11)
C2—C1—C6	118.3 (7)	N3A—C22A—H22C	108.8
C2—C1—Se1	122.5 (6)	C23A—C22A—H22C	108.8
C6—C1—Se1	119.2 (5)	N3A—C22A—H22D	108.8
C1—C2—C3	123.2 (8)	C23A—C22A—H22D	108.8
C1—C2—H2A	118.4	H22C—C22A—H22D	107.7
C3—C2—H2A	118.4	C22A—C23A—H23D	109.5
C2—C3—C4	119.3 (7)	C22A—C23A—H23E	109.5
C2—C3—H3A	120.4	H23D—C23A—H23E	109.5
C4—C3—H3A	120.4	C22A—C23A—H23F	109.5
C5—C4—C3	119.8 (7)	H23D—C23A—H23F	109.5
C5—C4—H4A	120.1	H23E—C23A—H23F	109.5
C3—C4—H4A	120.1	N3A—C24A—C25A	114.2 (9)
C4—C5—C6	120.2 (8)	N3A—C24A—H24C	108.7
C4—C5—H5A	119.9	C25A—C24A—H24C	108.7
C6—C5—H5A	119.9	N3A—C24A—H24D	108.7
C5—C6—C1	119.2 (7)	C25A—C24A—H24D	108.7
C5—C6—C7	116.6 (7)	H24C—C24A—H24D	107.6
C1—C6—C7	124.1 (7)	C24A—C25A—H25D	109.5
N1—C7—C6	117.5 (7)	C24A—C25A—H25E	109.5
N1—C7—H7A	121.3	H25D—C25A—H25E	109.5
C6—C7—H7A	121.3	C24A—C25A—H25F	109.5
O1—C8—N2	125.8 (6)	H25D—C25A—H25F	109.5
O1—C8—C9	129.0 (7)	H25E—C25A—H25F	109.5
N2—C8—C9	105.2 (6)	O2—C26—C27	114.7 (9)
C10—C9—C14	121.0 (7)	O2—C26—C31	125.3 (9)
C10—C9—C8	129.5 (7)	C27—C26—C31	120.0
C14—C9—C8	109.5 (6)	C26—C27—C28	120.0
C11—C10—C9	117.7 (8)	C26—C27—H27A	120.0
C11—C10—H10A	121.1	C28—C27—H27A	120.0
C9—C10—H10A	121.1	C29—C28—C27	120.0
C12—C11—C10	121.7 (8)	C29—C28—N4	121.3 (5)
C12—C11—H11A	119.2	C27—C28—N4	118.5 (5)
C10—C11—H11A	119.2	C28—C29—C30	120.0
C11—C12—C13	121.0 (8)	C28—C29—H29A	120.0
C11—C12—H12A	119.5	C30—C29—H29A	120.0
C13—C12—H12A	119.5	C29—C30—C31	120.0
C14—C13—C12	117.3 (8)	C29—C30—H30A	120.0
C14—C13—H13A	121.3	C31—C30—H30A	120.0
C12—C13—H13A	121.3	C30—C31—C26	120.0
C13—C14—C9	121.2 (7)	C30—C31—C15	120.8 (10)
C13—C14—C15	127.6 (7)	C26—C31—C15	119.1 (10)
C9—C14—C15	111.1 (6)	C28—N4—C34	120.1 (8)
N2—C15—C14	98.9 (6)	C28—N4—C32	120.2 (7)
N2—C15—C31A	111.4 (18)	C34—N4—C32	117.2 (9)
C14—C15—C31A	114.5 (18)	C33—C32—N4	104.4 (10)
N2—C15—C16A	111.5 (12)	C33—C32—H32A	110.9
C14—C15—C16A	108.3 (13)	N4—C32—H32A	110.9
C31A—C15—C16A	111.6 (18)	C33—C32—H32B	110.9
N2—C15—C16	110.2 (7)	N4—C32—H32B	110.9
C14—C15—C16	111.6 (8)	H32A—C32—H32B	108.9
N2—C15—C31	112.2 (10)	C32—C33—H33A	109.5
C14—C15—C31	113.3 (10)	C32—C33—H33B	109.5
C16—C15—C31	110.2 (10)	H33A—C33—H33B	109.5
C17—C16—C21	120.0	C32—C33—H33C	109.5
C17—C16—C15	119.6 (9)	H33A—C33—H33C	109.5
C21—C16—C15	120.4 (9)	H33B—C33—H33C	109.5
C16—C17—C18	120.0	N4—C34—C35	107.2 (9)
C16—C17—H17A	120.0	N4—C34—H34A	110.3
C18—C17—H17A	120.0	C35—C34—H34A	110.3
C19—C18—C17	120.0	N4—C34—H34B	110.3
C19—C18—H18A	120.0	C35—C34—H34B	110.3
C17—C18—H18A	120.0	H34A—C34—H34B	108.5
C18—C19—C20	120.0	C34—C35—H35A	109.5
C18—C19—N3	120.2 (5)	C34—C35—H35B	109.5
C20—C19—N3	119.8 (5)	H35A—C35—H35B	109.5
C21—C20—C19	120.0	C34—C35—H35C	109.5
C21—C20—H20A	120.0	H35A—C35—H35C	109.5
C19—C20—H20A	120.0	H35B—C35—H35C	109.5
O2—C21—C20	114.7 (8)	O2—C26A—C27A	115.1 (16)
O2—C21—C16	125.2 (8)	O2—C26A—C31A	124.9 (16)
C20—C21—C16	120.0	C27A—C26A—C31A	120.0
C19—N3—C22	120.9 (7)	C26A—C27A—C28A	120.0
C19—N3—C24	119.4 (7)	C26A—C27A—H27B	120.0
C22—N3—C24	118.2 (7)	C28A—C27A—H27B	120.0
N3—C22—C23	113.9 (10)	C29A—C28A—C27A	120.0
N3—C22—H22A	108.8	C29A—C28A—N4A	121.3 (6)
C23—C22—H22A	108.8	C27A—C28A—N4A	118.6 (6)
N3—C22—H22B	108.8	C28A—C29A—C30A	120.0
C23—C22—H22B	108.8	C28A—C29A—H29B	120.0
H22A—C22—H22B	107.7	C30A—C29A—H29B	120.0
C22—C23—H23A	109.5	C31A—C30A—C29A	120.0
C22—C23—H23B	109.5	C31A—C30A—H30B	120.0
H23A—C23—H23B	109.5	C29A—C30A—H30B	120.0
C22—C23—H23C	109.5	C30A—C31A—C26A	120.0
H23A—C23—H23C	109.5	C30A—C31A—C15	119.8 (19)
H23B—C23—H23C	109.5	C26A—C31A—C15	120.0 (19)
N3—C24—C25	114.1 (8)	C28A—N4A—C34A	119.9 (9)
N3—C24—H24A	108.7	C28A—N4A—C32A	120.3 (9)
C25—C24—H24A	108.7	C34A—N4A—C32A	117.2 (9)
N3—C24—H24B	108.7	C33A—C32A—N4A	104.5 (11)
C25—C24—H24B	108.7	C33A—C32A—H32C	110.9
H24A—C24—H24B	107.6	N4A—C32A—H32C	110.9
C24—C25—H25A	109.5	C33A—C32A—H32D	110.9
C24—C25—H25B	109.5	N4A—C32A—H32D	110.9
H25A—C25—H25B	109.5	H32C—C32A—H32D	108.9
C24—C25—H25C	109.5	C32A—C33A—H33D	109.5
H25A—C25—H25C	109.5	C32A—C33A—H33E	109.5
H25B—C25—H25C	109.5	H33D—C33A—H33E	109.5
C17A—C16A—C21A	120.0	C32A—C33A—H33F	109.5
C17A—C16A—C15	118.9 (16)	H33D—C33A—H33F	109.5
C21A—C16A—C15	121.1 (16)	H33E—C33A—H33F	109.5
C16A—C17A—C18A	120.0	N4A—C34A—C35A	107.5 (10)
C16A—C17A—H17B	120.0	N4A—C34A—H34C	110.2
C18A—C17A—H17B	120.0	C35A—C34A—H34C	110.2
C19A—C18A—C17A	120.0	N4A—C34A—H34D	110.2
C19A—C18A—H18B	120.0	C35A—C34A—H34D	110.2
C17A—C18A—H18B	120.0	H34C—C34A—H34D	108.5
C18A—C19A—C20A	120.0	C34A—C35A—H35D	109.5
C18A—C19A—N3A	120.1 (6)	C34A—C35A—H35E	109.5
C20A—C19A—N3A	119.9 (6)	H35D—C35A—H35E	109.5
C21A—C20A—C19A	120.0	C34A—C35A—H35F	109.5
C21A—C20A—H20B	120.0	H35D—C35A—H35F	109.5
C19A—C20A—H20B	120.0	H35E—C35A—H35F	109.5
			
C7—N1—N2—C8	33.2 (11)	C31A—C15—C16A—C17A	−174 (2)
C7—N1—N2—C15	−156.7 (7)	N2—C15—C16A—C21A	−120.5 (13)
C6—C1—C2—C3	−1.9 (11)	C14—C15—C16A—C21A	131.7 (12)
Se1—C1—C2—C3	178.4 (6)	C31A—C15—C16A—C21A	5 (3)
C1—C2—C3—C4	1.2 (12)	C21A—C16A—C17A—C18A	0.0
C2—C3—C4—C5	0.0 (12)	C15—C16A—C17A—C18A	179 (2)
C3—C4—C5—C6	−0.4 (12)	C16A—C17A—C18A—C19A	0.0
C4—C5—C6—C1	−0.3 (11)	C17A—C18A—C19A—C20A	0.0
C4—C5—C6—C7	−178.4 (7)	C17A—C18A—C19A—N3A	180 (2)
C2—C1—C6—C5	1.4 (10)	C18A—C19A—C20A—C21A	0.0
Se1—C1—C6—C5	−178.8 (5)	N3A—C19A—C20A—C21A	−180 (2)
C2—C1—C6—C7	179.4 (6)	C19A—C20A—C21A—C16A	0.0
Se1—C1—C6—C7	−0.9 (9)	C19A—C20A—C21A—O2	−167 (3)
N2—N1—C7—C6	−177.8 (6)	C17A—C16A—C21A—C20A	0.0
C5—C6—C7—N1	175.5 (7)	C15—C16A—C21A—C20A	−178 (2)
C1—C6—C7—N1	−2.5 (10)	C17A—C16A—C21A—O2	166 (3)
N1—N2—C8—O1	−8.7 (12)	C15—C16A—C21A—O2	−13 (2)
C15—N2—C8—O1	−178.8 (7)	C26A—O2—C21A—C20A	−176 (2)
N1—N2—C8—C9	169.5 (7)	C26A—O2—C21A—C16A	17 (3)
C15—N2—C8—C9	−0.6 (8)	C18A—C19A—N3A—C22A	5 (3)
O1—C8—C9—C10	−3.6 (14)	C20A—C19A—N3A—C22A	−175.4 (17)
N2—C8—C9—C10	178.3 (8)	C18A—C19A—N3A—C24A	175.9 (17)
O1—C8—C9—C14	178.3 (8)	C20A—C19A—N3A—C24A	−4 (2)
N2—C8—C9—C14	0.3 (8)	C19A—N3A—C22A—C23A	74 (2)
C14—C9—C10—C11	0.5 (12)	C24A—N3A—C22A—C23A	−97 (2)
C8—C9—C10—C11	−177.3 (8)	C19A—N3A—C24A—C25A	−78 (3)
C9—C10—C11—C12	1.6 (13)	C22A—N3A—C24A—C25A	94 (3)
C10—C11—C12—C13	−3.4 (15)	C21—O2—C26—C27	−177.5 (12)
C11—C12—C13—C14	2.8 (13)	C21—O2—C26—C31	4.7 (15)
C12—C13—C14—C9	−0.7 (12)	O2—C26—C27—C28	−177.9 (13)
C12—C13—C14—C15	175.9 (8)	C31—C26—C27—C28	0.0
C10—C9—C14—C13	−1.0 (12)	C26—C27—C28—C29	0.0
C8—C9—C14—C13	177.2 (7)	C26—C27—C28—N4	−175.1 (11)
C10—C9—C14—C15	−178.1 (7)	C27—C28—C29—C30	0.0
C8—C9—C14—C15	0.1 (9)	N4—C28—C29—C30	175.0 (11)
C8—N2—C15—C14	0.7 (8)	C28—C29—C30—C31	0.0
N1—N2—C15—C14	−170.8 (6)	C29—C30—C31—C26	0.0
C8—N2—C15—C31A	121.4 (18)	C29—C30—C31—C15	−176.6 (18)
N1—N2—C15—C31A	−50.0 (18)	O2—C26—C31—C30	177.7 (14)
C8—N2—C15—C16A	−113.1 (14)	C27—C26—C31—C30	0.0
N1—N2—C15—C16A	75.4 (14)	O2—C26—C31—C15	−5.7 (10)
C8—N2—C15—C16	−116.3 (9)	C27—C26—C31—C15	176.6 (17)
N1—N2—C15—C16	72.2 (9)	N2—C15—C31—C30	−54.6 (13)
C8—N2—C15—C31	120.4 (10)	C14—C15—C31—C30	56.3 (13)
N1—N2—C15—C31	−51.0 (11)	C16—C15—C31—C30	−177.8 (9)
C13—C14—C15—N2	−177.3 (8)	N2—C15—C31—C26	128.8 (8)
C9—C14—C15—N2	−0.5 (8)	C14—C15—C31—C26	−120.3 (9)
C13—C14—C15—C31A	64 (2)	C16—C15—C31—C26	5.6 (15)
C9—C14—C15—C31A	−118.9 (18)	C29—C28—N4—C34	17.8 (13)
C13—C14—C15—C16A	−61.1 (15)	C27—C28—N4—C34	−167.2 (9)
C9—C14—C15—C16A	115.8 (13)	C29—C28—N4—C32	179.1 (9)
C13—C14—C15—C16	−61.4 (12)	C27—C28—N4—C32	−5.9 (12)
C9—C14—C15—C16	115.5 (9)	C28—N4—C32—C33	−73.2 (14)
C13—C14—C15—C31	63.7 (13)	C34—N4—C32—C33	88.6 (12)
C9—C14—C15—C31	−119.4 (11)	C28—N4—C34—C35	63.9 (14)
N2—C15—C16—C17	51.8 (11)	C32—N4—C34—C35	−98.0 (12)
C14—C15—C16—C17	−57.0 (11)	C21A—O2—C26A—C27A	167 (2)
C31—C15—C16—C17	176.2 (12)	C21A—O2—C26A—C31A	−15 (3)
N2—C15—C16—C21	−129.8 (7)	O2—C26A—C27A—C28A	178 (3)
C14—C15—C16—C21	121.4 (8)	C31A—C26A—C27A—C28A	0.0
C31—C15—C16—C21	−5.4 (13)	C26A—C27A—C28A—C29A	0.0
C21—C16—C17—C18	0.0	C26A—C27A—C28A—N4A	−176 (2)
C15—C16—C17—C18	178.4 (11)	C27A—C28A—C29A—C30A	0.0
C16—C17—C18—C19	0.0	N4A—C28A—C29A—C30A	176 (2)
C17—C18—C19—C20	0.0	C28A—C29A—C30A—C31A	0.0
C17—C18—C19—N3	178.9 (10)	C29A—C30A—C31A—C26A	0.0
C18—C19—C20—C21	0.0	C29A—C30A—C31A—C15	174 (4)
N3—C19—C20—C21	−178.9 (10)	O2—C26A—C31A—C30A	−178 (3)
C26—O2—C21—C20	179.1 (11)	C27A—C26A—C31A—C30A	0.0
C26—O2—C21—C16	−4.4 (18)	O2—C26A—C31A—C15	7 (2)
C19—C20—C21—O2	176.8 (15)	C27A—C26A—C31A—C15	−174 (4)
C19—C20—C21—C16	0.0	N2—C15—C31A—C30A	−51 (2)
C17—C16—C21—O2	−176.4 (16)	C14—C15—C31A—C30A	60 (2)
C15—C16—C21—O2	5.2 (13)	C16A—C15—C31A—C30A	−176.5 (17)
C17—C16—C21—C20	0.0	N2—C15—C31A—C26A	123.3 (17)
C15—C16—C21—C20	−178.4 (11)	C14—C15—C31A—C26A	−125.6 (16)
C18—C19—N3—C22	4.3 (15)	C16A—C15—C31A—C26A	−2 (3)
C20—C19—N3—C22	−176.7 (9)	C29A—C28A—N4A—C34A	−2 (2)
C18—C19—N3—C24	169.8 (9)	C27A—C28A—N4A—C34A	173.9 (16)
C20—C19—N3—C24	−11.2 (13)	C29A—C28A—N4A—C32A	−163.3 (16)
C19—N3—C22—C23	78.1 (13)	C27A—C28A—N4A—C32A	13 (2)
C24—N3—C22—C23	−87.6 (13)	C28A—N4A—C32A—C33A	−99 (2)
C19—N3—C24—C25	−70.6 (18)	C34A—N4A—C32A—C33A	99 (2)
C22—N3—C24—C25	95.3 (17)	C28A—N4A—C34A—C35A	−120 (2)
N2—C15—C16A—C17A	61 (2)	C32A—N4A—C34A—C35A	42 (3)
C14—C15—C16A—C17A	−47 (2)		

Symmetry code: (i) *y*+1/2, *x*−1/2, −*z*+1.

### Hydrogen-bond geometry (Å, º)

**Table:**

*D*—H···*A*	*D*—H	H···*A*	*D*···*A*	*D*—H···*A*
C2—H2*A*···Se1^i^	0.95	2.82	3.436 (8)	124
C7—H7*A*···O1	0.95	2.43	2.940 (9)	114
C7—H7*A*···O1^ii^	0.95	2.63	3.391 (10)	137
C12—H12*A*···O1^iii^	0.95	2.56	3.447 (10)	156
C33*A*—H33*E*···Se1^i^	0.98	3.04	4.00 (3)	168

Symmetry codes: (i) *y*+1/2, *x*−1/2, −*z*+1; (ii) *y*+1/2, *x*−1/2, −*z*+2; (iii) *y*+1, −*x*+1, −*z*+2.

### Percentage contributions of interatomic contacts to the Hirshfeld surface for the title compound

**Table:**

Contact	Percentage contribution
H···H	68.1
C···H/H···C	21.2
O···H/H···O	8.7
N···H/H···N	1.6
Se···H/H···Se	0.4

### Comparison of selected (X-ray and DFT) bond lengths and angles (Å, °)

**Table:**

	X-ray	B3LYP/6-31G(d)
Se1—C1	1.939 (7)	1.941
Se1—Se1'	2.3517 (17)	2.356
O1—C8	1.222 (9)	1.224
O2—C21	1.315 (12)	1.374
O2—C26	1.349 (13)	1.368
N1—C7	1.280 (9)	1.291
N1—N2	1.380 (8)	1.353
N2—C8	1.378 (10)	1.392
N2—C15	1.507 (9)	1.513
C1—C6	1.415 (11)	1.42
C19—N3	1.426 (7)	1.383
C22—N3	1.446 (13)	1.464
C24—N3	1.466 (12)	1.461
N4—C28	1.423 (10)	1.39
N4—C32	1.543 (14)	1.461
N4—C34	1.481 (13)	1.462
C1—Se1—Se1'	103.5 (2)	102.711
C26—O2—C21	119.4 (9)	119.241
C7—N1—N2	121.3 (6)	122.983
N1—N2—C15	115.5 (5)	116.391
C2—C1—Se1	122.5 (6)	121.361
C6—C1—Se1	119.2 (5)	119.262
O1—C8—N2	125.8 (6)	126.543
C8—N2—C15	115.3 (5)	114.337
O1—C8—C9	129.0 (7)	128.268
N2—C8—C9	105.2 (6)	105.19
C19—N3—C22	120.9 (7)	120.635
C19—N3—C24	119.4 (7)	120.99
C22—N3—C24	118.2 (7)	118.187
C28—N4—C32	120.2 (7)	120.875
C28—N4—C34	120.1 (8)	120.772
C32—N4—C34	117.2 (9)	117.917
C1—Se1—Se1'—C1'	-88.9 (6)	-73.195

Symmetry code: (i) 1/2 + *y*, *x* - 1/2, 1 - *z*.
